# Microbial diversity within the digestive tract contents of Dezhou donkeys

**DOI:** 10.1371/journal.pone.0226186

**Published:** 2019-12-13

**Authors:** Guiqin Liu, Gerelchimeg Bou, Shaofeng Su, Jingya Xing, Honglei Qu, Xinzhuang Zhang, Xisheng Wang, Yiping Zhao, Manglai Dugarjaviin

**Affiliations:** 1 College of Animal Science, Inner Mongolia Key Laboratory of Equine Genetics, Breeding and Reproduction, Equine Research Center, Inner Mongolia Agricultural University, Hohhot, China; 2 College of Agronomy, Liaocheng University, Shandong Engineering Technology Research Center for Efficient Breeding and Ecological Feeding of Black Donkey, Shandong Donkey Industry Technology Collaborative Innovation Center, Liaocheng, Shandong Province, China; 3 National Engineering Research Center for Gelatin-based Traditional Chinese Medicine, Dong-E-E-Jiao Co. Ltd., Dong-E Country, Shandong Province, China; University of Maine, UNITED STATES

## Abstract

Gastrointestinal microbiota has significant impact on the nutrition and health of monogastric herbivores animals including donkey. However, so far the microbiota in different gastrointestinal compartments of healthy donkey has not been described. Therefore, we investigated the abundance and function of microbiota at different sites of the gastrointestinal tract (GIT) (foregut: stomach, duodenum, jejunum and ileum; hindgut: cecum, ventral colon, dorsal colon, and rectum) of healthy adult donkeys mainly based on 16S rRNA gene sequencing and phylogenetic investigation of communities by reconstruction of unobserved states (PICRUSt) analysis. Collectively, our results showed that donkey has a rich, diverse and multi-functional microbiota along the GIT. In general, the richness and diversity of the microbiota are much higher in the hindgut relative to that in the foregut; at phylum level, the Firmicutes is dominant in the foregut while both Firmicutes and Bacteroides are abundant in the hindgut; at the genus level, *Lactobacillus* was dominant in the foregut while *Streptococcus* was more dominant in the hindgut. Our further PICRUSt analysis showed that varying microbiota along the GIT is functionally compatible with the corresponding physiological function of different GIT sites. For example, the microbes in the foregut are more active at carbohydrate metabolism, and in the hindgut are more active at amino acid metabolism. This work at the first time characterized the donkey digestive system from the aspects of microbial composition and function, provided an important basic data about donkey healthy gastrointestinal microbiota, which may be utilized to evaluate donkey health and also offer clues to further investigate donkey digestive system, nutrition, even to develop the microbial supplements.

## Introduction

Different animal species have different characteristics of their digestive system. It is largely related to their unique anatomical structure and diet type. Therefore, different input and process result in different output, for example the amount of soluble carbohydrate or fiber reaching the large intestine varies among different species [1]. All of these inevitably give rise to a unique gastrointestinal tract (GIT) microbiota for each species. Among all digestive types, monogastric herbivorous animals have been reported susceptible to the change of microbial communities in their digestive tracts [2]. Unfortunately, only sparse data have been published about healthy GIT microbiota of this kind of animals, even blank for donkey.

As we know, donkey as an an *Equus* animal has a well-developed hindgut structure, which has a potential length and volume over 4.5 meters and 110 liters, and about 15.95 times the volume of its foregut. Microbial fermentation always play important role in equines, that along with small amounts of other organic compounds, such as methane, carbon dioxide, lactate, alcohol, and lots of volatile fatty acid were also produced in several parts of their gastrointestinal tract[3]. On the aspect of energy provision, Bergman (1971) reported that approximately 60–70% of equine energy needs are provided by organic acids from microbial fermentation in their large intestine [4]. On the aspect of fiber digestion, anaerobic fermentation by cellulosic bacteria can breakdown the structural carbohydrates in their cecum and large colon [3, 5]. However, although microorganisms are very important for equine digestion and metabolism, so far limited studies have been reported the microbiota in rectal samples of healthy horses [6, 7], and only one study analyzed the fecal microbiota composition in donkey [8]. A systematical investigation on healthy donkey GIT microbiota is needed.

Studies in human and mice have shown that anatomic regions of GIT must function correctly and in concert with the other region to maintain the health and nutrition. These differential functions of different regions more or less rely on microorganism in it. The difference in the diet [9], pH [10], oxygen tension [11] and so on many factors leads to the differential microbiota at different GIT regions. For example, due to the unique and strong digestive function of rumen, it is inhabited by a dense, distinctive consortium of microorganisms according to the reports in cows, sheep, yak, reindeer and sika deer [12–16]. A large variation of microbial populations along the GIT was seen within horses [17]. From these former studies, we could clearly found that healthy GIT microbiota should respond to the function of different GIT sections. Thus, a study on GIT microbiota function would improve our knowledge on gradient digestion and metabolism process in animal’s GIT and the role of microbe in it.

Aimed to the current blank state of GIT microbiota study in donkey, we decided to characterize and compare the microbiota compositions and functions at the different GIT regions mainly based on 16s rRNA gene sequencing and phylogenetic investigation of communities by reconstruction of unobserved states (PICRUSt) analysis results of luminal contents from stomach, duodenum, jejunum and ileum, cecum, ventral colon, dorsal colon, and rectum of five healthy China Dezhou donkeys.

## Materials and methods

### Experimental duration and venue

This study was carried out from May 26 to July 25, 2018 (60 days) at the National Breeding Center of Dezhou Black Donkeys, Dong-E-E-Jiao Co., Ltd. (http://www.dongeejiao.com/) (Shandong Province, China).

### Experimental animals

The Dezhou male donkeys (n = 5) used in this study were bred on the same farm and with an average age of 2 years ± 3 months, an average body weight of 215 ± 10 kg. All donkeys were fed in individual stalls (3×4 m) with a feeder (1.0 m long) and an automatic water dispenser. The entire feeding process was carried out under outdoor natural lighting and by a specially trained person. Supplementary feeding was administered at 1.5% of the bodyweight of each experimental donkey twice daily (07:00 and 17:00). In addition, all donkeys were fed with roughage (bean straw) four times a day (07:00, 11:00, 17:00, and 22:00) and with water throughout the day.

The five donkeys at the end of the feeding were fasted for 12 h before slaughter. Donkeys were stunned by an electrical stunner (about 280 V) and were then slaughtered at Dong-E-E-Jiao Co., Ltd. During feeding and before slaughter, all donkeys were regularly examined by a veterinarian to confirm that they were healthy and without any metabolic or gastrointestinal disorder. These animal experiments were approved by the Animal Welfare Committee of Liaocheng University, and all procedures were conducted in accordance with the guidelines of the China Animal Protection Association.

### Sample collection

The contents from various regions of the GIT of the five healthy Dezhou donkeys were collected after slaughter. Sampling of the gastrointestinal contents in different donkeys was conducted in a manner as consistent as possible. The sampling was as follows: stomach contents were collected from the pylorus; duodenal contents were collected at the site 10 cm after the gastroduodenal junction; jejunal contents were collected at the site 10 cm after the duodeno-jejunal junction; ileal contents were collected at the site 10 cm before the ileo-cecal orifice; cecal contents were collected from the tip of the cecum; ventral colonic contents were collected from the middle of the ventral colon; dorsal colonic contents were collected from the middle of the dorsal colon; and rectal contents were collected near the anus (S1 Fig).

The gastrointestinal contents were collected, handled, and stored as aseptically as possible in order to prevent contamination in any manner. The contents were stored in 50 mL sterile cryopreservation tubes that were immediately placed in liquid nitrogen, and then transported to laboratory to store in a -80°C freezer.

### Total DNA extraction, purification, and library construction

Genomic DNA was extracted using a QIAamp DNA Stool Mini Kit (QIAGEN, Valencia, CA) following the manufacturer’s instructions. The DNA was then checked by gel electrophoresis to determine its purity and quantity. Equal amounts of sample DNA were placed in a centrifuge tube and diluted to 1 ng/μL with sterile water. The diluted genomic DNA was used as the DNA template, and the V3–V4 region of the bacterial 16S ribosomal RNA (rRNA) gene was amplified using primers 341F (5-’CCTAYGGGRBGCASCAG-3’) and 806R (5’-GGACTACNNGGGTATCTAAT-3’) [9] for all samples.

The Ion Plus Fragment Library Kit (48 reactions, Thermo Fisher Scientific, Waltham, MA, USA) was used for library construction. The constructed library was subjected to Qubit quantitation and library testing, followed by sequencing using an Ion S5^TM^ XL system (Thermo Fisher Scientific). Total DNA extraction, PCR, and sequencing using an Ion S5^TM^ XL system were completed by Novogene Co. Ltd. (Beijing, China).

### Bioinformatics analysis

According to the methodology described by Martin [18], the Cutadapt (V1.9.1, http://cutadapt.readthedocs.io/en/stable/) server was used to remove low-quality reads. Different sample data were separated from the obtained reads according to the barcodes. Initial quality control was carried out by removing barcode and primer sequences to obtain raw data (raw reads), which were required to remove chimeric sequences using the following website (http://www.drive5.com/usearch/manual/chimera_formation.html). The read sequences were aligned with species annotation databases through the UCHIME Algorithm (http://www.drive5.com/usearch/manual/uchime_algo.html) [19]in order to identify chimeric sequences. The chimeric sequences were then removed to obtain the final valid data (clean reads) [20].

All clean reads were clustered using Uparse v7.0.1001 software (http://drive5.com/uparse/) [21]. By default, the sequences were clustered into operational taxonomic units (OTUs) with 97% identity. The representative sequences of OTUs were subjected to species annotation (threshold was defaulted as 0.8–1) using the Mothur method and SILVA SSU rRNA database (http://www.drive5.com/usearch/manual/uchime_algo.html) [22] to obtain taxonomic information and to count the microbiota compositions of different samples at various taxonomic levels: kingdom, phylum, class, order, family, genus, and species. Rapid multiple sequence alignments were performed using MUSCLE Version 3.8.31 [23] (http://www.drive5.com/muscle/) to obtain the phylogenetic relationships of all representative OTUs. Finally, the data from different samples were normalized, and the sample with the least amount of data was used as the standard for data normalization. Subsequently, alpha diversity was analyzed based on the data after normalization. Data were made publicly available at the NCBI Sequence Read Archive under the accession number PRJNA556136.

The average abundance and alpha diversity of the microbiota were calculated using Qiime (Version 1.9.1). The evaluation indices selected for this analysis included Chao1, Shannon, and Observed species. Chao1 and Observed species were used to calculate the community abundance, and the ecological diversity of each sample was assessed by the Shannon diversity index. The abundance and diversity of species in the samples were counted at two taxonomic levels: phylum and genus. R software (Version 2.15.3) was used to prepare a dilution curve and analyze the differences in alpha diversity indices between groups.

PICRUSt analysis include three steps: first, "Closed-reference OTU picking" was performed on the 16S rRNA gene sequences obtained by sequencing, and through comparison with the Greengenes database, the "nearest neighbor of the reference sequence" of each sequencing sequence was found and classified as the reference OTU; second, according to the rRNA copy number of the nearest neighbor of the reference sequence, the obtained OTU abundance matrix was corrected; the third, according to the functional genes data of KEGG (Kyoto Encyclopedia of Genes and Genomes) or eggNOG (evolutionary genealogy of genes: Non-supervised Orthologous Groups) and other genes corresponding to the "nearest neighbor of reference sequence", the overall metabolic function of the bacterial community was predicted by conversion. In this work, the function predicted of all samples were analyzed using PICRUSt. The closed OTU-table obtained by QIIME (Version 1.9.1) was compared with eggnog and KEGG databases to obtain different database function prediction information. The specific analysis steps were based on the online analysis platform (http://picrust.github.io/picrust/) [24].

### Data analysis

SPSS19.0 (IBM SPSS, Chicago, IL) software was used for statistical analysis of numbers of metabolic functional genes among GIT sites. For this case one-way ANOVA was conducted and data was showed as the mean ± SD, with significance level at P < 0.05.

## Results

### OTUs distribution along the donkey GIT

For 8 GIT sites’ content samples of 5 donkeys, 16s rRNA gene sequencing obtained a total of 3,106,234 effective sequences, with an average of 77,656 ± 7,582 sequences per sample (S1 Table). Then, all effective sequences underwent species annotation at different taxonomic levels, which yielded a total of 7,459 OTUs belonging to 35 phyla, 55 classes, 118 orders, 222 families, and 401 genera.

**Fig 1:**
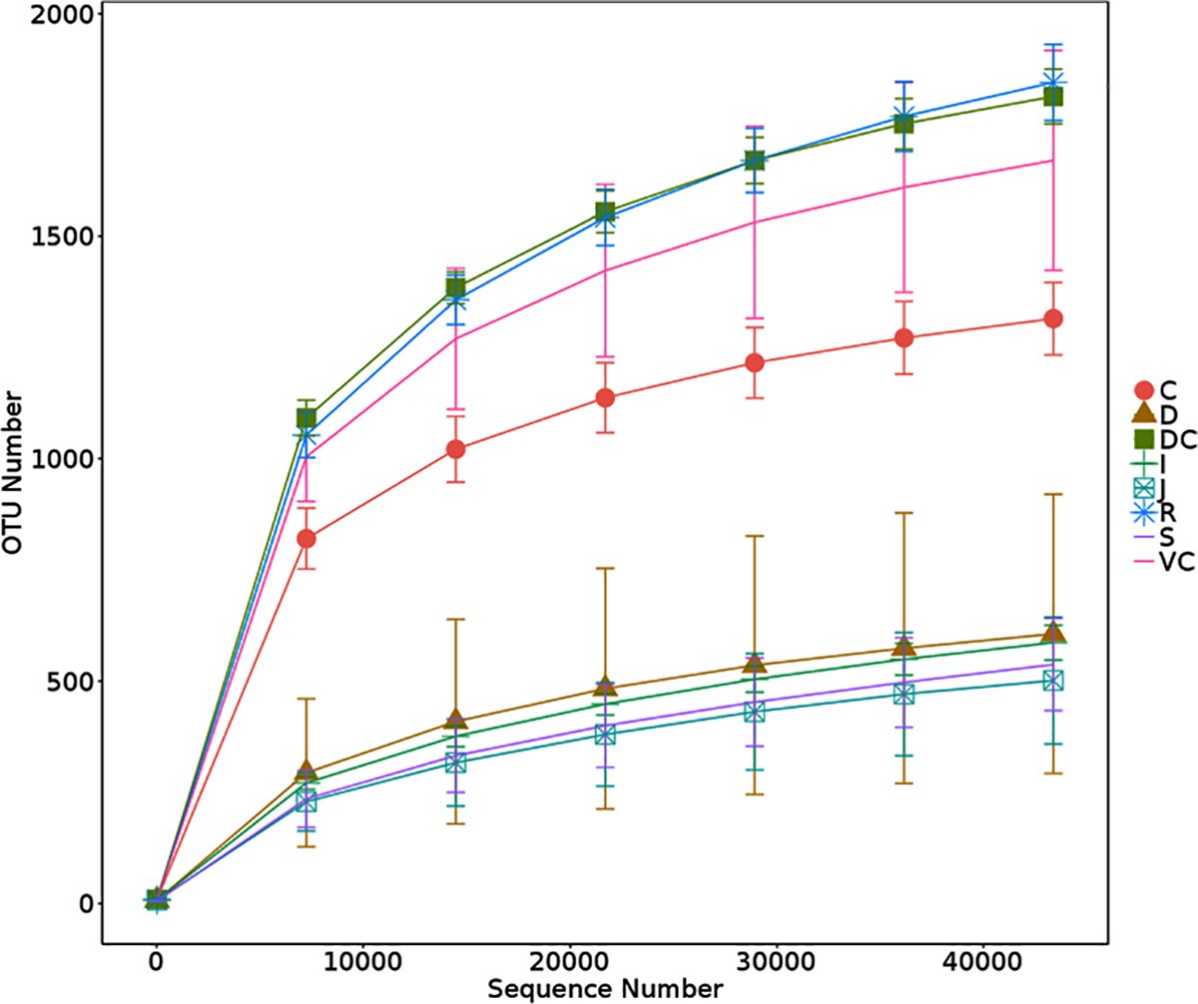
Rarefaction curves of samples. In the rarefaction curve, the X-axis is the number of sequencing strips which were randomly extracted from a sample, and the Y-axis is the number of OTU constructed based on the number of sequencing strips, which is used to reflect the sequencing depth. Different samples are represented by different color curves. S = stomach, D = duodenum, J = jejunum, I = ileum, C = cecum, VC = ventral colon, DC = dorsal colon, R = rectal. These abbreviations are same in all figures.

Rarefaction curves directly reflect the rationality of sequencing quantity and the richness of species in samples. When the curve flattens out, it means that the number of sequences is reasonable, and more data can only produce a few new species. Fig 1 shows the rarefaction curves of samples collected from different parts of the GIT. The rarefaction curves of different samples eventually came to be flat, indicating that the majority of gastrointestinal microbes were covered in every sample under the sequencing depth (reads = 43,384) of this study.

**Fig 2:**
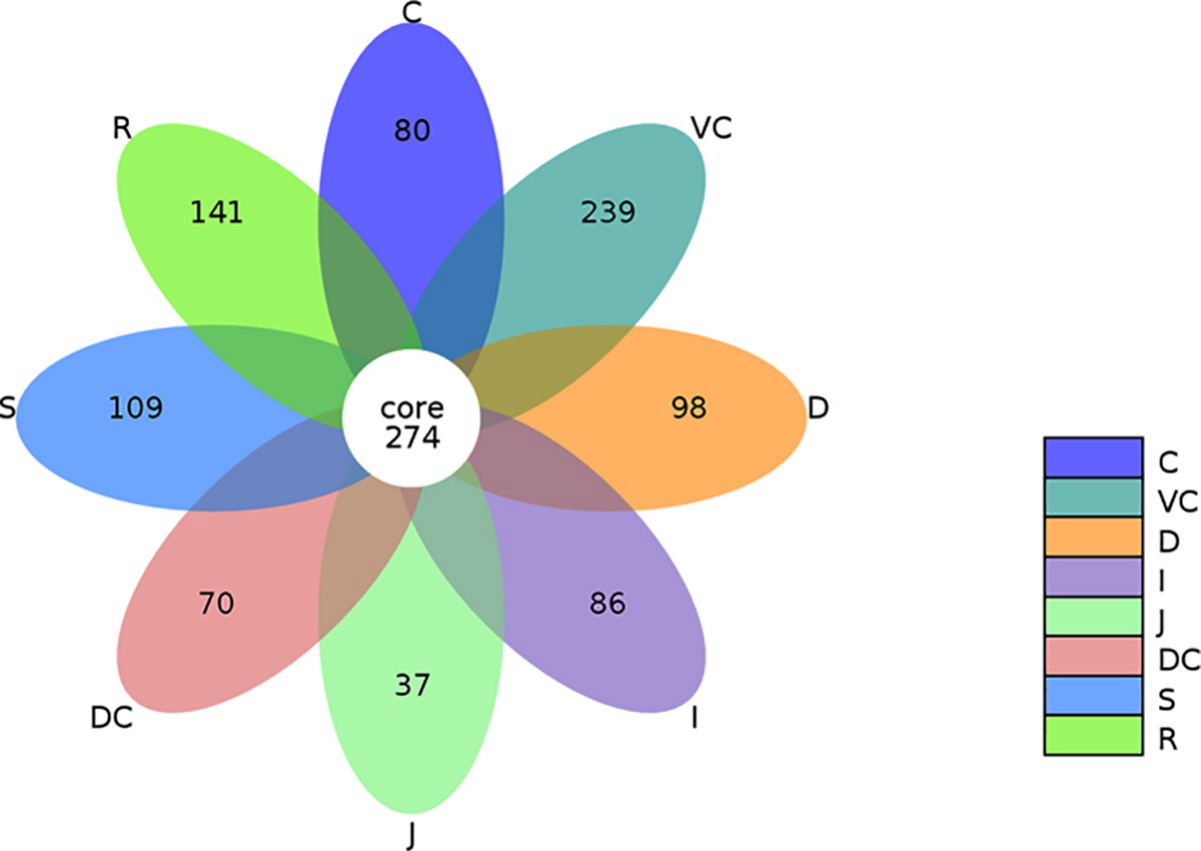
Flower Diagram of OTUs distribution in GIT. Each petal in the diagram represents a sample, and different colors represent different samples. The core number in the middle represents the total number of mutual OTUs in all samples, and the number on the petal represents the number of unique OTU in this sample.

To show the distribution of both the common and unique OTUs among samples, a flower diagram was drawn (Fig 2). The microbial species from intestinal samples showed high richness and diversity, that the number of common OTUs in all samples was 274, and plenty of unique OTUs were found in stomach (109), duodenum (98), jejunum (37), ileum (86), cecum (80), ventral colon (239), dorsal colon (70), and rectum (141) (Fig 2).

### Microbiota richness and diversity along the donkey GIT

Further we analyzed the bacterial richness and diversity in the different GIT regions of donkeys when the sequencing depth was 43,384 (Table 1). As shown in Table 1, significant differences in the species richness (Observed species: 1171, 501–1845; Chao1: 1343, 71–2105) and species diversity (Shannon: 6.6, 3.87–8.69) were found among GIT sites. All indices showed that microbiota along the GIT could be divided into 3 levels: lower in foregut (stomach, duodenum, jejunum, ileum), medium in cecum and higher in hindgut except cecum.

**Table 1:** Comparison of the diversity indices of bacterial communities in different GIT sites of donkeys at a sequencing depth of 4,3384.

Items	Chao1	Shannon	Observed species
Stomach	741.56±80.95^C^	3.87±0.40^D^	537.25±103.61^C^
Duodenum	71.35±318.04^C^	4.42±0.68^CD^	606.25±313.52^C^
Jejunum	641.78±93.83^C^	4.53±0.36^CD^	501.33±123.54^C^
Ileum	769.34±92.74^C^	4.64±0.33^C^	586.5±39.12^C^
Cecum	1416.30±90.73^B^	8.11±0.47^B^	1314.80±90.98^B^
Ventral colon	1856.95±280.68^A^	8.61±0.38^A^	1670.40±276.55^A^
Dorsal colon	1982.87±58.56^A^	8.69±0.24^A^	1814.25±61.70^A^
Rectum	2105.08±166.22^A^	8.37±0.51^A^	1845.80±97.75^A^

Notes: (1) Analysis of different samples under the threshold of 97% identity. (2) Different uppercase letters means the significance at P < 0.05, and same letters in the superscripts represent P > 0.05.

### Relative abundance of gastrointestinal microbiota at the phylum and the genus level along the donkey GIT

To contrast similarities and difference in the community structures in different GIT sites, we analyzed the relative abundance of gastrointestinal microbiota at the phylum and genus level respectively.

**Fig 3:**
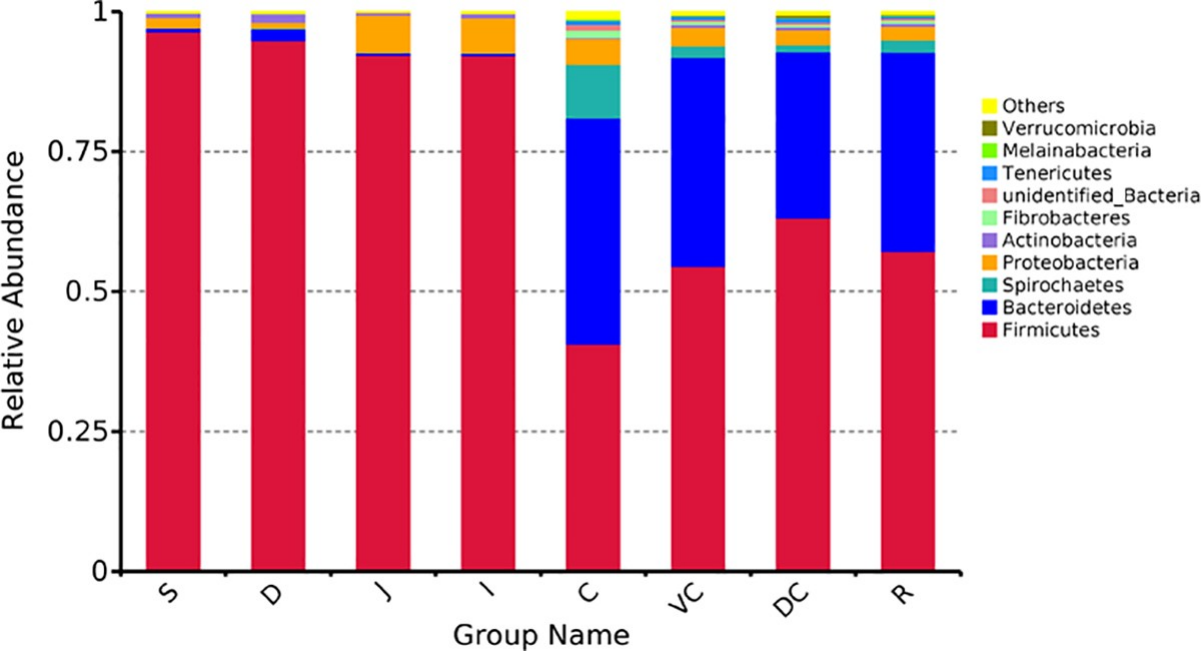
The relative abundance of bacterial communities at the phylum level in the different site GIT luminal contents of five donkeys (top 10 most abundant phyla).

At the phylum level, as shown in Fig 3 and S2 Table, Firmicutes is dominant in the foregut, while both Firmicutes and Bacteroides are abundant (both accounting for >40%) in the hindgut.

**Fig 4:**
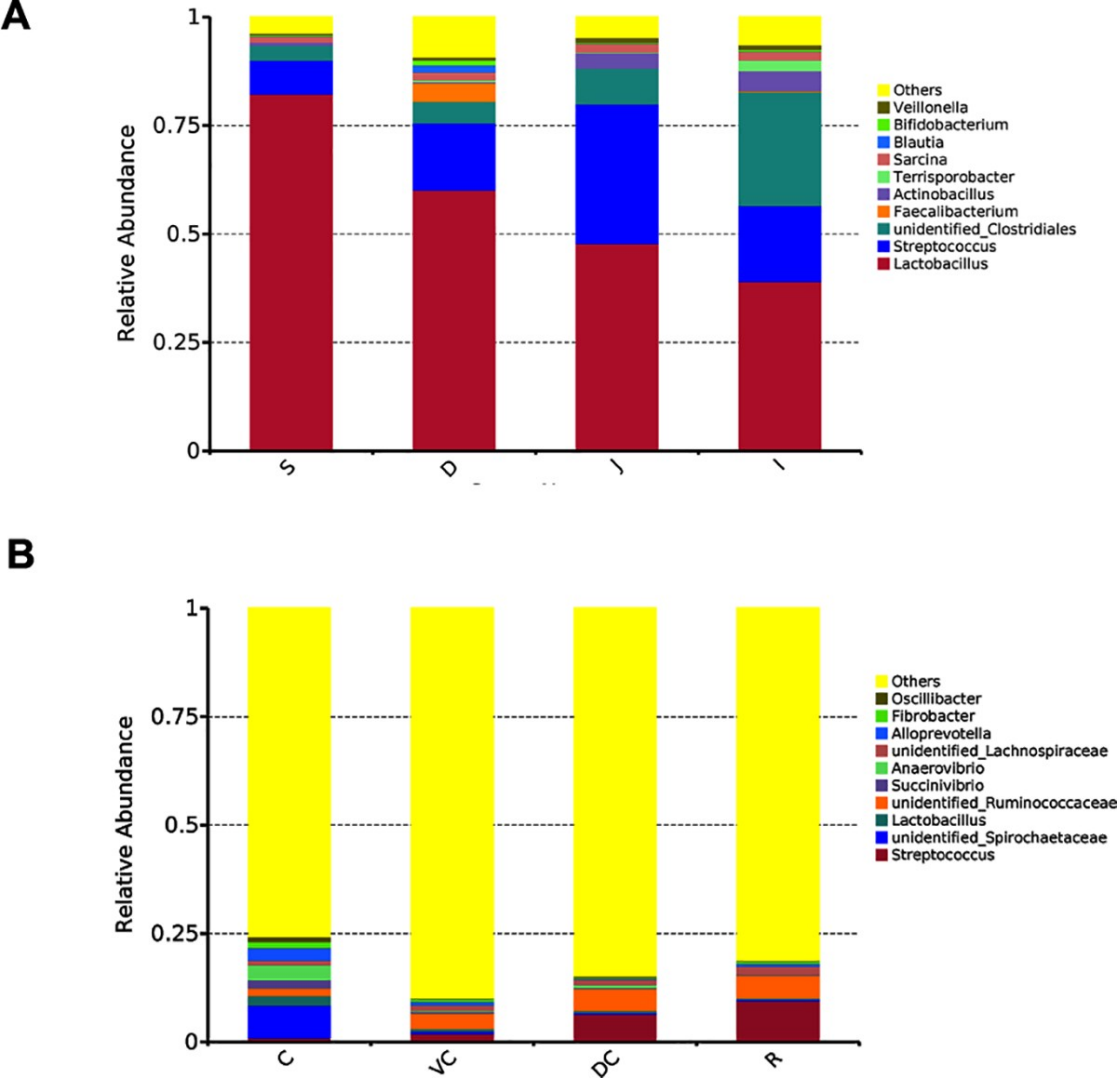
The relative abundance of bacterial communities at genus level in the different site GIT luminal contents of five donkeys. Bar charts showing the relative abundance detected in foregut (A, top 10 genera for foregut) and hindgut (B, top 10 genera for hindgut) samples.

At the genus level, after regrouping the relatively less abundant genera (< 1%) as “others”, we selected the top 10 abundant genera among the 401 genera of gastrointestinal microbes respectively for foregut and hindgut. As shown in Fig 4 and S3 Table, *Lactobacillus* (accounting for up to 82.27%) was dominant genus in the foregut, *Streptococcus* was the most abundant one (1.86–9.45%) in the hindgut, especially in rectum and dorsal colon, while unidentified Spirochaetaceae (7.63%) was higher in the cecum than in the other gastrointestinal regions (P<0.05). More than 75% of “others” genera in all parts of hindgut also implied their rich diversity of microbiota.

### Functional prediction of microbiota along the GIT using PICRUSt approach

**Fig 5:**
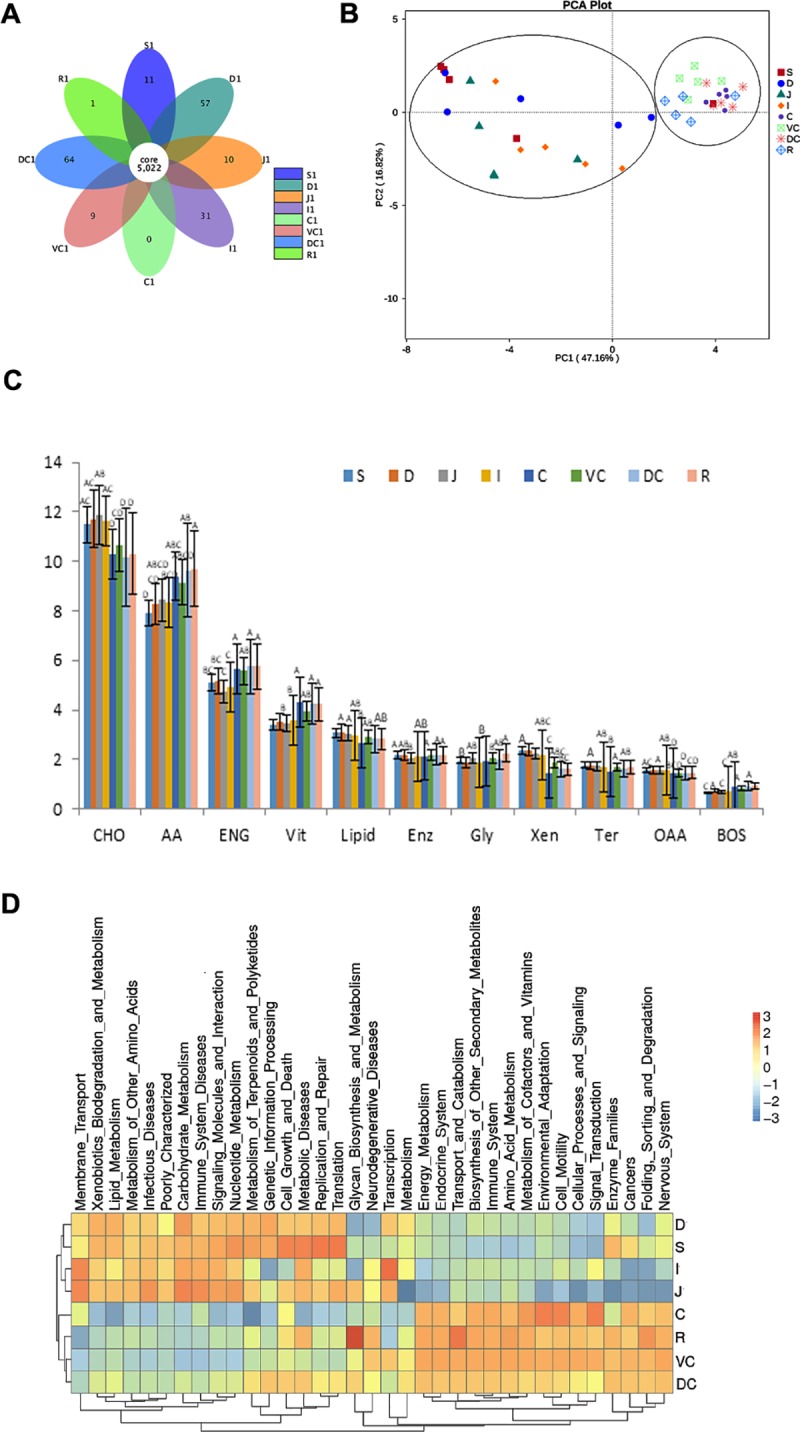
Analysis of the functional genes of microbiota in GIT at KO level. (A): Flower diagram of function gene at KEGG KO level, each petal in the diagram represents a sample, and different colors represent different samples, and the core number in the middle represents the total number of mutual functional gene in all samples, and the number on the petal represents the unique genes in this sample. (B): Principal component analysis (PCA) based on KO level. On the aspects of functional genes, GIT sites are clustered into 2 big groups that respectively included in 2 circles. (C): Statistical analysis of the metabolic functional genes’ numbers along donkey GIT. Different uppercase letters means the significance at P < 0.05. CHO: Carbohydrate; AA: Amino Acid; ENG: Energy; Vit: Cofactors and Vitamins; Enz: Enzyme Families; Gly: Glycan Biosynthesis and Metabolism; Xen: Xenobiotics Biodegradation and Metabolism; Ter: Terpenoids and Polyketides; OAA: Other Amino Acids; Bos: Biosynthesis of Other Secondary Metabolites. (D): Heatmap showing the expressional abundances of genes related to various metabolisms.

Based on the 16S rRNA of bacteria and the OTU informations, we used KEGG, PICRUSt approach to predict the gene functions of GIT microbiota. As shown in Fig 5A, although mictobiota from all GIT sites share a large number (5022) of functional genes, there were a total number of 183 functional genes that specifically present in different sites. Among them, the cecum and rectum are lack of unique functional genes.

Then principle component analysis (PCA) on these functional genes at the KEGG orthology (KO) level showed that all GIT sites could be clustered into 2 groups, foregut group and hindgut group (Fig 5B), in which the samples from hindgut showed higher homogeneity, especially cecum samples.

After relating the functional genes with metabolism process, we found that in terms of metabolism process of amino acid, energy, cofactors and vitamins, and biosynthesis of other secondary metabolite, the number of the related functional genes were significantly higher in hindgut than in foregut (P < 0.05), whereas in terms of carbohydrate and other amino acids metabolism process, the number of the related functional genes function genes were significantly higher in foregut than in hindgut (P < 0.05) (Fig 5C). At last, we analyzed the gene expression level of top 35 functional genes in different GIT sites and showed it in a heatmap (Fig 5D). The expression profiles of different GIT sites confirmed the metabolism bias of different GIT sites seen in Fig 5B and 5C. From the heatmap we could see that there was an obvious difference in the functional gene expression profiles between the foregut and hindgut.

## Discussion

This is the first work reporting the microbiota and their function along the GIT of healthy donkeys. Previous research on gastrointestinal microbes has mainly focused on cecum [25], and fecal samples [26]of horses. However, there is a great significance to understand the full picture of health microbial communities in the entire GIT of donkey.

Our results show that there are great variations of microbiota along the donkeys GIT, not only the diversity but also the abundance. These results agree with lots of previous findings in horses [27], mice [28], human [29] and dog [30]. This shift may correspond to the physiological function of the digestive tract, which physical and chemical parameters were various along the GIT, such as pH, diet etc. Furthermore, the present data shows that donkeys have two distinct regions along the GIT in microbial communities, which agree with previous findings of horses [31], that the difference between foregut and hindgut are significant (P < 0.05), however, there are no difference among GIT sites within foregut or within hindgut (except cecum) (P >0.05). In equine, the foregut is chiefly responsible for food digestion and absorption, and the hindgut is related to microbial fermentation [32]. The slight gastric fermentation has been confirmed in stomach and small intestine of equine, but still large intestine is the most important part for fermentation of forage [3, 33]. Our study shows that the species and the distribution of microbes vary among different gastrointestinal regions, and their significant difference reflected at Shannon indices (alpha-diversity) was consistent with the previous results of healthy horses [17]. Microbial diversity and abundance indices of the hindgut were also significantly higher than that of the foregut, suggesting greater complexity of microbial communities in the hindgut.

Moreover, here we shows that Firmicutes was the most common phylum in all gastrointestinal regions (> 50% relative abundance), except the cecum, where both Firmicutes and Bacteroidetes were abundant (Fig 3). The relative abundance of Proteobacteria was high in the foregut, and the relative abundance of Bacteroidetes was high in the hindgut. The phylum Proteobacteria includes many pathogenic bacteria, which might indicate an increased chance of Dezhou donkeys contracting gastrointestinal infection from the microbes in the foregut. Previous studies have shown that Firmicutes and Bacteroidetes are the dominant microbial communities in the GIT of rodents, swine, horses, and cattle [34–36]. Firmicutes is the main microbial phylum that promotes fiber decomposition in the gastrointestinal tract of herbivores [37], and Bacteroidetes is the main microbial phylum that metabolizes carbohydrates in herbivores [38]. Thus, this indicates that in terms of phylum level bacterial composition Dezhou donkeys has a good basis of the crude feed tolerance. In addition, the larger proportion of Bacteroidetes in the hindgut of Dezhou donkeys suggests that the hindgut plays a role in energy supply, which energy metabolism genes was highly significant higher in hindgut (Fig 5C). And it is consistent with previous results in horse that the large intestine supplies 60%-70% energy needs [4].

When we look at the bacterial composition at the genus level, our study shows that *Lactobacillus* was the main genus in the foregut. *Lactobacillus* is comprised of mostly mutualistic bacteria that have a strong tolerance to acid and can selectively kill pathogenic microbes, deconstruct bile acids and produce free bile acids to promote fat metabolism [39]. In consistent with it, the number and expression of genes related to lipid metabolism were found extremely higher in the foregut (Fig 5C and 5D). In the hindgut, the relative abundances of most of the genera are < 1%, and unidentified *Spirochaetaceae* is the most common in the cecum. The unidentified *Spirochaetaceae* family is closely involved in fiber degradation, such as cellobiose, by producing cellulase, an enzyme found in typical cellulose-metabolizing strains [40]. Studies by Patra et al. [41] and Zhao et al. [42] have shown that the number of *Spirochaetaceae* is closely related to cellulose digestion and utilization. The cellulolytic bacteria inhabit the cecum more often than the colon [3]. The function genes abundant in Fig 5C and 5D also show that cecum has a strong cellulolytic microbial basis in donkey. However, one study reported that *Ruminococcus flavefaciens* was the predominant species in the equine cecum [43]. This discrepancy maybe caused by the feeding conditions. In contrast, *Streptococcus* had the highest abundance in donkey dorsal colon and rectum (Fig 4B), which can be induced by arginine and subjected to carbon catabolite repression [44]. Some reports indicated that *Streptococcus* was an important cause of infectious diseases. These results also clearly show us that fecal microbiota cannot represent other GIT microbiota [45]. Noteworthy, at both the phylum and genus levels, the microbial communities of various sections were similar within the hindgut than within the foregut, which might be caused by complicated physiological condition along the foregut due to the gradient effect of gastric acid.

As we know, clear differences in function and metabolism bias exist between the foregut and hindgut. This point is confirmed by our PICRUSt functional prediction analysis of donkey intestinal microbes. The functional genes in the stomach and duodenum, genes in the ileum and jejunum, genes in the dorsal colon and the ventral colon respectively show similar bias to metabolism and this result is consistent with the analysis of microbial diversity along the GIT. In detail our study show that the metabolism process related to amino acid, energy, enzyme families, cofactors/vitamins, and biosynthesis of other amino acids are more active in hindgut than in foregut, while carbohydrate and other amino acids metabolism process are more active in foregut than in hindgut. Our result that the majority of metabolisms are active in donkey hindgut may well explain why previous study found that large intestine supplies 60%-70% energy needs in equine [4].

## Conclusions

The present study mainly based on 16S rRNA gene high-throughput sequencing and PICRUSt approach provided new insight into bacterial communities and function along the GIT of healthy Dezhou donkeys. The following conclusions could be drawn: (1) Microbial communities in the donkey GIT are abundant in diversity and population. (2) Because of the different microbial compositions, the predicted microbiota functions of different GIT sections are varied. (3) Based on the microbial compositions and functions, doneky GIT could be divided into 2 big groups: foregut and hindgut. (4) The microbiota in rectum (feces) samples is only similar to it in colon, hard to represent the microbiota in other intestinal compartments.

## Supporting information

S1 Fig The anatomy of the GIT of Dezhou donkeys. Foregut and hindgut was divided from the ileocecal valves.
(PDF)

S1 Table Statistics of taxonomic and OTU numbers of samples. (PDF)

S2 Table The list of bacterial communities relative abundance at phyla of every samples. (PDF)

S3 Table The list of bacterial communities relative abundance at genus level of every samples. (PDF)
