# Supplementary material for: Microbial diversity within the digestive tract contents of Dezhou donkeys
Source: PLoS One. 2019 Dec 13;14(12):e0226186. doi: 10.1371/journal.pone.0226186 (PMC6910686; doi:10.1371/journal.pone.0226186)
Supplement: S1 Fig — Foregut and hindgut was divided from the ileocecal valves. (PDF) [file pone.0226186.s001.pdf]

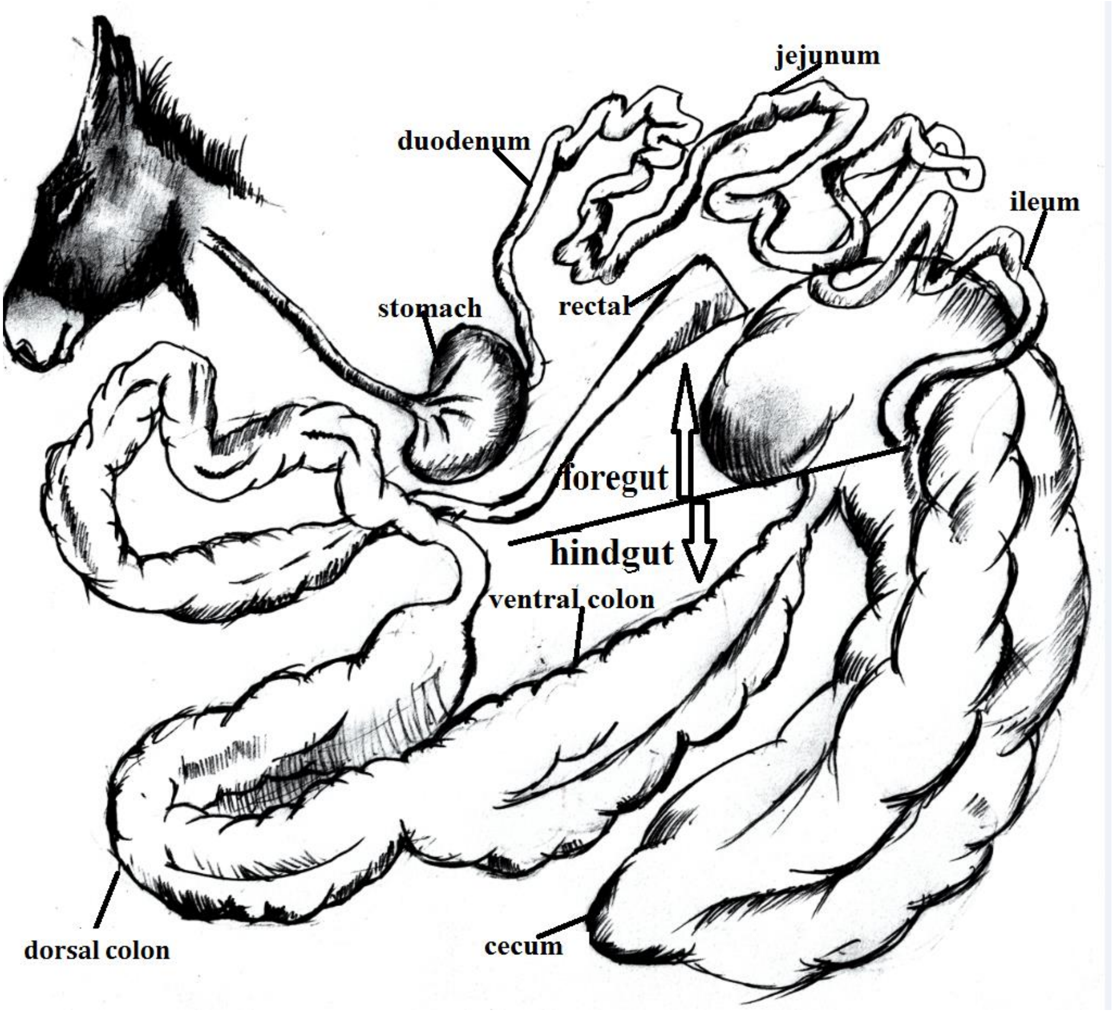

S1 Figure. Sampling of the GIT of Dezhou donkeys. Foregut and hindgut was divided by the ileocecal valves.
