# Supplementary material for: Microbial diversity within the digestive tract contents of Dezhou donkeys
Source: PLoS One. 2019 Dec 13;14(12):e0226186. doi: 10.1371/journal.pone.0226186 (PMC6910686; doi:10.1371/journal.pone.0226186)
Supplement: S1 Table — (PDF) [file pone.0226186.s002.pdf]

S1 Table. Statistics of taxonomic and OUT numbers of samples

Sample_Name	Total_tag	Taxon_Tag	Unclassified_Tag	Unique_Tag	OTU_num
S1.1	80337	71619	0	8718	608
S1.2	84020	75981	0	8039	763
S1.3	80264	73061	0	7203	580
S1.4	80253	68116	0	12137	834
S1.5	80239	64263	0	15976	1238
D1.1	54291	47583	2	6706	1276
D1.2	65665	59133	3	6529	447
D1.3	63855	53771	8	10076	1511
D1.4	80163	65241	0	14922	756
D1.5	81588	72814	1	8773	454
J1.1	70793	60213	0	10580	855
J1.2	80499	71961	0	8538	554
J1.3	70854	61892	0	8962	607
J1.4	70854	61892	0	8962	607
J1.5	80216	68064	0	12152	515
I1.1	84542	72043	0	12499	740
I1.2	64844	57780	0	7064	699
I1.3	80168	70572	0	9596	730
I1.4	80156	66684	0	13472	831
I1.5	80250	67690	0	12560	636
C1.1	80119	61631	0	18488	1541
C1.2	80127	65071	0	15056	1458
C1.3	80215	64975	0	15240	1549
C1.4	80183	60212	0	19971	1531
C1.5	80044	61995	0	18049	1317
VC1.1	80061	63162	0	16899	2112
VC1.2	80318	65104	0	15214	2023
VC1.3	66116	52072	0	14044	1964
VC1.4	80024	65866	0	14158	1918
VC1.5	87441	79903	0	7538	2366
DC1.1	80135	67763	0	12372	2785
DC1.2	80128	63938	0	16190	2371
DC1.3	80236	64338	0	15898	1652
DC1.4	80134	65108	0	15026	1916
DC1.5	80213	65401	0	14812	1608
R1.1	95786	76310	0	19476	2238
R1.2	80224	67508	0	12716	2073
R1.3	78128	60241	0	17887	1978
R1.4	80441	66416	0	14025	2039
R1.5	62350	43387	0	18963	1856
Total	3106274	64769.35		515486	53536
