# Supplementary material for: Microbial diversity within the digestive tract contents of Dezhou donkeys
Source: PLoS One. 2019 Dec 13;14(12):e0226186. doi: 10.1371/journal.pone.0226186 (PMC6910686; doi:10.1371/journal.pone.0226186)
Supplement: S2 Table — (PDF) [file pone.0226186.s003.pdf]

S2 Table. The list of bacterial communities relative abundance at phyla level of every samples.

Taxonomy	Firmicutes	Proteobacteria	Bacteroidetes	Spirochaetes	Fusobacteria	unidentified_Bacteria	Actinobacteria	Fibrobacteres	Melainabacteria	Tenericutes	Others
S1.1	0.982428758	0.010044306	0.004908871	0.000453127	0.00020139	5.03E-05	0.001183164	0.000176216	0	5.03E-05	0.000503
S1.2	0.970244688	0.007954889	0.013191018	0.00020139	0.000553821	0.000125868	0.006998288	0	0	0.000100695	0.000629
S1.3	0.982579801	0.01354345	0.001862854	0.0004783	0.00020139	7.55E-05	0.000956601	2.52E-05	7.55E-05	5.03E-05	0.000151
S1.4	0.921030108	0.044431578	0.007728325	0.000125868	0.002366328	0.00020139	0.020919343	0	2.52E-05	5.03E-05	0.003122
S1.5	0.657562179	0.028169369	0.287257074	0.007627631	0	0.00319706	0.004027792	0.002894975	0.00067969	0.004052965	0.004531
D1.1	0.914409425	0.020063438	0.046571342	0.006394119	0.000377605	0.000730037	0.003373276	0.002240459	0.000906253	0.001434901	0.003499
D1.2	0.987790756	0.001938375	0.002391501	0.000151042	0.00020139	7.55E-05	0.004959219	2.52E-05	0	5.03E-05	0.002417
D1.3	0.788893364	0.050246702	0.117661867	0.013191018	0.00387675	0.002013896	0.010648474	0.003398449	0.002139764	0.002743933	0.005186
D1.4	0.897366831	0.019383748	0.033279629	0.000125868	0.000881079	0.000730037	0.047477595	0	0	0	0.000755
D1.5	0.994436613	0.001686638	0.00163629	0.000100695	2.52E-05	5.03E-05	0.001913201	5.03E-05	0	0	0.000101
J1.1	0.792518377	0.192830531	0.006092035	0.00183768	0.000654516	0.000302084	0.004128487	0.000427953	0.000352432	0.00020139	0.000655
J1.2	0.988697009	0.002190112	0.002064243	0.000251737	0.000100695	0	0.006217904	2.52E-05	2.52E-05	5.03E-05	0.000378
J1.3	0.986683113	0.00735072	0.000906253	0.000226563	0.000125868	0.000100695	0.003952271	2.52E-05	0	0.000151042	0.000478
J1.4	0.985953076	0.007753499	0.001107643	0.000176216	0.000125868	0.000125868	0.004027792	0	0	7.55E-05	0.000655
J1.5	0.986960024	0.005966167	0.003121539	2.52E-05	5.03E-05	0.000176216	0.003448797	0.000151042	0	0	0.000101
I1.1	0.921583929	0.063890847	0.003146712	0.000427953	0.0004783	7.55E-05	0.003096365	0	0	7.55E-05	0.007225
I1.2	0.96760145	0.018553016	0.005563387	0.00067969	0.000629342	7.55E-05	0.005689256	7.55E-05	2.52E-05	0.00020139	0.000906
I1.3	0.866503877	0.12523915	0.004078139	0.000276911	0.000302084	0.000125868	0.002341154	0.000100695	0	5.03E-05	0.000982
I1.4	0.932106535	0.045942	0.001888027	0.000276911	0.000805558	0.000276911	0.017822979	0.000100695	0.00020139	7.55E-05	0.000503
I1.5	0.957557144	0.027514852	0.002441849	7.55E-05	0.000251737	5.03E-05	0.011302991	7.55E-05	0	2.52E-05	0.000705
C1.1	0.417002316	0.017999194	0.422238445	0.109505589	7.55E-05	0.00231598	0.000402779	0.009717048	0.000427953	0.011957507	0.008358
C1.2	0.336295439	0.023487061	0.581965562	0.030460175	0.000176216	0.004455745	0.001283859	0.008634579	2.52E-05	0.003373276	0.009843
C1.3	0.473668311	0.110462189	0.256947941	0.096918739	2.52E-05	0.014651093	0.00067969	0.004883698	0.010975733	0.014399356	0.016388

C1.4	0.450483335	0.047930722	0.431955493	0.028698016	0.000327258	0.008458363	0.00387675	0.006998288	0.000327258	0.004984392	0.01596
C1.5	0.359958715	0.032373376	0.320712919	0.215587554	0	0.015909777	0.000906253	0.038515759	0.000805558	0.005261303	0.009969
VC1.1	0.63075219	0.012083375	0.313941194	0.007929715	7.55E-05	0.003801228	0.003297755	0.006318598	0.001132816	0.005160608	0.015507
VC1.2	0.598655724	0.019811701	0.332947337	0.022480113	0.000327258	0.008785621	0.001082469	0.001183164	0.00067969	0.004808176	0.009239
VC1.3	0.58964354	0.052738898	0.314444668	0.009037358	0.000100695	0.004934045	0.007703152	0.006494814	0.000730037	0.004304702	0.009868
VC1.4	0.709218608	0.023537408	0.224624912	0.011655422	0.000100695	0.003423623	0.007098983	0.001309032	0.000553821	0.013845534	0.004632
VC1.5	0.995871513	0.001585943	0.00067969	5.03E-05	0.000251737	0	0.001132816	0	0	0	0.000428
DC1.1	0.508735273	0.026835163	0.411237539	0.025224046	5.03E-05	0.001888027	0.001736985	0.014474877	0.000427953	0.003851576	0.005538
DC1.2	0.583601853	0.02185077	0.348605377	0.007677978	0.000100695	0.002945323	0.010321216	0.000730037	0.000402779	0.009213574	0.01455
DC1.3	0.458815829	0.044884704	0.427877354	0.042921156	2.52E-05	0.001938375	0.001283859	0.00850871	0.000704864	0.007627631	0.005412
DC1.4	0.558579196	0.042568724	0.359757326	0.01749572	0.000276911	0.002265633	0.00435505	0.004178834	0.000327258	0.00667103	0.003524
DC1.5	0.619398852	0.028647669	0.31915215	0.008307321	7.55E-05	0.009314268	0.004204008	0.002844628	0.000276911	0.003222233	0.004556
R1.1	0.524544356	0.015079045	0.395730541	0.028144195	7.55E-05	0.003625013	0.002013896	0.011907159	0.003373276	0.004531266	0.010976
R1.2	0.484442654	0.009062531	0.45038264	0.035545262	7.55E-05	0.001762159	0.002114591	0.003348102	0.000830732	0.004934045	0.007502
R1.3	0.53655221	0.049743228	0.382917128	0.008659752	7.55E-05	0.003448797	0.005815124	0.006268251	0.000629342	0.002290807	0.0036
R1.4	0.616906656	0.020994865	0.299843923	0.037810895	7.55E-05	0.002618065	0.005613735	0.007098983	0.000730037	0.004052965	0.004254
R1.5	0.696103111	0.027288289	0.249773437	0.003851576	0	0.003499144	0.006167556	0.003549491	0.001132816	0.005462693	0.003172
