# Supplementary material for: Microbial diversity within the digestive tract contents of Dezhou donkeys
Source: PLoS One. 2019 Dec 13;14(12):e0226186. doi: 10.1371/journal.pone.0226186 (PMC6910686; doi:10.1371/journal.pone.0226186)
Supplement: S3 Table — (PDF) [file pone.0226186.s004.pdf]

S3 Table. The list of bacterial communities relative abundance at genus level of every samples.

Taxonomy	Lactobacillus	Streptococcus	Actinobacillus	unidentified_Clostridiales	Weissella	Sarcina	unidentified_Spirochaetaceae	Stenotrophomonas	Faecalibacterium	Fusobacterium	Others
S1.1	0.887045615	0.011378512	0.007778673	0.064268452	5.03E-05	0.00619273	0.000302084	0.000176216	5.03E-05	0.000100695	0.022656329
S1.2	0.940766287	0.008961837	0.002718759	0.001510422	0	7.55E-05	5.03E-05	0.000327258	0.001057295	0.000151042	0.04438123
S1.3	0.951314067	0.015481825	0.008357668	0.002416675	7.55E-05	0.004178834	0.00020139	0.0004783	2.52E-05	0.000151042	0.017319505
S1.4	0.511630249	0.278698016	0.010522606	0.067843118	0.001888027	0.043500151	5.03E-05	0.000830732	0.000100695	0.002064243	0.082871816
S1.5	0.004052965	0.073129594	0.000352432	0.009440137	0	0.000100695	0.00163629	0.013593797	0.000100695	0	0.897593394
D1.1	0.64593193	0.064545363	0.010925385	0.064369147	2.52E-05	0.060844829	0.004808176	0.000176216	0.000352432	0.000276911	0.147744437
D1.2	0.900614238	0.038918538	0.000151042	0.027464505	2.52E-05	0.000151042	2.52E-05	5.03E-05	5.03E-05	5.03E-05	0.032499245
D1.3	0.3897392	0.045841305	0.005487866	0.099058504	0.000176216	0.044305709	0.007980062	0.00803041	0.001560769	7.55E-05	0.397744437
D1.4	0.115874534	0.328013292	0.000629342	0.051656429	0.004707482	0.004506092	5.03E-05	0.000151042	0.171810492	0.000402779	0.322198167
D1.5	0.744537307	0.185807069	0.000327258	0.054400363	7.55E-05	0.004883698	2.52E-05	0	5.03E-05	0	0.009893264
J1.1	0.463472963	0.071493304	0.134402376	0.091279831	2.52E-05	0.074035847	0.001006948	0.000151042	0.000402779	0.000226563	0.163503172

J1.2	0.787735374	0.083224247	0.000956601	0.090625315	7.55E-05	0.000402779	0.000125868	0.000100695	7.55E-05	2.52E-05	0.036652905
J1.3	0.099184372	0.810542745	0.001384553	0.063286678	0.000654516	0.002794281	0.000151042	0.000302084	0	0.000100695	0.021599033
J1.4	0.098781593	0.810215487	0.001460075	0.063764978	0.000654516	0.00251737	0.000100695	0.000402779	2.52E-05	0.000100695	0.021976639
J1.5	0.561650388	0.323280636	0.002567717	0.085036754	0.00020139	0.003121539	2.52E-05	0.000125868	5.03E-05	5.03E-05	0.02388984
I1.1	0.241189206	0.093797201	0.049340449	0.458337529	0	0.068975934	0.00020139	0.000226563	0.000830732	0.000327258	0.086773739
I1.2	0.76538113	0.053896889	0.014097271	0.097598429	5.03E-05	0.000352432	0.000427953	7.55E-05	0.006973114	0.000327258	0.060819656
I1.3	0.491038163	0.12224348	0.099108851	0.208841003	5.03E-05	0.001686638	0.00020139	0.000654516	0.000402779	0.000125868	0.075646964
I1.4	0.064671231	0.434019736	0.022706676	0.281542644	0.00938979	0.004531266	0.000100695	0.000377605	0.00020139	0.000604169	0.181854798
I1.5	0.348076729	0.383143691	0.020441043	0.191848756	5.03E-05	0.015028698	5.03E-05	0.000100695	5.03E-05	0.000100695	0.04110865
C1.1	0.003549491	0.00135938	0.00231598	0.007929715	2.52E-05	0.000125868	0.075596617	0.00020139	0.002643238	2.52E-05	0.906227973
C1.2	0.008961837	0.002592891	0.000629342	0.014726614	0	5.03E-05	0.019081663	0.000151042	0.002240459	0.000100695	0.951465109
C1.3	0.001988722	0.002366328	0.001434901	0.002190112	0	0.000100695	0.081311046	0.002643238	0.001711811	0	0.906253147
C1.4	0.009440137	0.030913302	0.001434901	0.018099889	0	0.000176216	0.013467929	0.002164938	0.001988722	0.000176216	0.92213775

C1.5	0.079372671	0.011630249	0.007325546	0.011252643	0	7.55E-05	0.191823583	0.000251737	0.001762159	0	0.696505891
VC1.1	0.004329876	0.076880475	0.000125868	0.009314268	0	0.000176216	0.00115799	0.003272581	0.000100695	0	0.90464203
VC1.2	0.005387171	0.006117209	5.03E-05	0.025375088	0	0.00020139	0.00898701	0.000604169	0.000578995	0	0.95269862
VC1.3	0.003121539	0.027816937	0.000151042	0.011353338	0	0.000176216	0.002341154	0.02552613	0.000151042	0	0.929362602
VC1.4	0.005085087	0.147316484	7.55E-05	0.015431477	7.55E-05	0.000352432	0.001913201	0.011126775	5.03E-05	2.52E-05	0.818547981
VC1.5	0.974650086	0.010849864	0.000352432	0.00435505	2.52E-05	0.003046018	2.52E-05	0	0	7.55E-05	0.006620683
DC1.1	0.004304702	0.051958514	0.000100695	0.008156278	0	5.03E-05	0.003096365	0.00367536	7.55E-05	0	0.928582217
DC1.2	0.003171886	0.016866378	0.000151042	0.010296043	5.03E-05	0.000100695	0.001384553	0.00231598	0.000100695	0	0.96556238
DC1.3	0.00319706	0.004883698	0.000302084	0.010447085	2.52E-05	0.000226563	0.025651999	0.021221428	0.001183164	0	0.932861746
DC1.4	0.002819454	0.017923673	0.000100695	0.015834256	2.52E-05	0.000125868	0.002139764	0.028345585	0	7.55E-05	0.932610009
DC1.5	0.015355956	0.001585943	0.000327258	0.026054778	0	0.000125868	0.002869802	0.006268251	0.000780385	0	0.946631759
R1.1	0.00299567	0.029125969	0.000251737	0.005689256	0	0.000125868	0.003625013	0.002064243	7.55E-05	2.52E-05	0.956021549
R1.2	0.002391501	0.013845534	0.000125868	0.004757829	5.03E-05	0.000100695	0.008256973	0.001460075	0.000226563	0	0.968784614

R1.3	0.002945323	0.001107643	0.000151042	0.00898701	2.52E-05	0.000151042	0.002794281	0.026382036	0.00020139	0	0.95725506
R1.4	0.00319706	0.18172893	5.03E-05	0.006041688	0	0.00020139	0.00251737	0.012486154	5.03E-05	0	0.793726714
R1.5	0.00735072	0.246878461	0.00020139	0.011680596	0	2.52E-05	0.001736985	0.013996576	0.000100695	0	0.718029403
